# Head and Neck Necrotizing Fasciitis: Abbreviated SOFA Score Associated With Death and Infection Spread

**DOI:** 10.1002/oto2.68

**Published:** 2023-08-09

**Authors:** Kelly L. Vittetoe, Samuel R. Johnson, Teresa A. Benvenuti, Jonathan G. Schoenecker, Stephanie N. Moore‐Lotridge, Sarah L. Rohde

**Affiliations:** ^1^ Department of Otolaryngology Vanderbilt University Medical Center Nashville Tennessee USA; ^2^ Vanderbilt University School of Medicine Nashville Tennessee USA; ^3^ Department of Orthopaedics Vanderbilt University Medical Center Nashville Tennessee USA; ^4^ Department of Pharmacology Vanderbilt University Nashville Tennessee USA; ^5^ Department of Pathology, Microbiology, and Immunology Vanderbilt University Medical Center Nashville Tennessee USA; ^6^ Division of Pediatric Orthopaedics Monroe Carell Jr. Children's Hospital Nashville Tennessee USA; ^7^ Vanderbilt Center for Bone Biology Vanderbilt University Medical Center Nashville Tennessee USA

**Keywords:** descending necrotizing mediastinitis, necrotizing fasciitis, prognostic score

## Abstract

**Objective:**

Describe features unique to head and neck (H&N) necrotizing fasciitis (NF) compared to other anatomic regions and specify a prognostic score associated with death and descending necrotizing mediastinitis (DNM).

**Study Design:**

Retrospective cohort.

**Setting:**

Tertiary care, level 1 trauma center.

**Methods:**

A single‐institution database identified 399 confirmed cases of NF between 2006 and 2021, 33 of which involved the H&N. Patients with confirmed H&N NF were sorted into cohorts based on clinical outcomes, with the “poor” outcomes group defined by death and/or DNM.

**Results:**

Thirty‐three patients with H&N NF were included. Compared to NF of other regions, patients with H&N NF had a significantly lower mortality rate (6.06% vs 20.8%, *p* = .041) and significantly lower rates of obesity (27.3% vs 63.7%, *p* < .001) and hypertension (42.4% vs 60.9%, *p* = .038). Within the H&N group, there were 2 deaths (6.06%) and 8 cases of DNM (24.2%). Diabetes was associated with poor outcomes (*p* = .047), as was an abbreviated sequential organ failure assessment score for necrotizing fasciitis (nfSOFA) of 2 or greater (*p* = .015).

**Conclusion:**

H&N NF is unique among other forms of NF, with a lower mortality rate and lower rates of obesity and hypertension in affected patients. Within the H&N cohort, worse outcomes were associated with diabetes as well as a nfSOFA score of 2 or greater. Timely surgical debridement alongside broad‐spectrum antibiotics remains the mainstay of treatment for NF; however, this simple prognostic score may play a role during the early stages of care for patients with H&N NF.

Necrotizing fasciitis (NF) of the head and neck (H&N) is a rare, rapidly progressive infection that requires timely surgical debridement for effective management.^1^ Despite recent advances in treatment modalities for NF, morbidity and mortality from this condition remain high.^2^ An especially feared and highly morbid complication of H&N NF is descending necrotizing mediastinitis (DNM), which involves spread of necrotizing infection from the neck to the thorax into the mediastinum.^3^ The mortality rate for DNM is substantial, estimated to be 30% to 40% across studies.^4^, ^5^, ^6^, ^7^, ^8^, ^9^

Numerous acute phase reactants^2^, ^10^, ^11^ and prognostic scores such as Laboratory Risk Indicator for Necrotizing Fasciitis (LRINEC)^12^, ^13^ and sequential organ failure assessment (SOFA)^14^, ^15^ have been utilized clinically to track infection progression and distinguish NF from cellulitis.^2^, ^10^, ^11^, ^12^, ^13^, ^14^, ^15^, ^16^, ^17^, ^18^, ^19^, ^20^, ^21^, ^22^ A prior study by Jabbour and colleagues has shown SOFA scores to be higher among nonsurvivors compared to survivors of necrotizing fasciitis of all anatomic regions.^23^ Multiple others have reported that comorbidities such as diabetes, cirrhosis, hypertension, heart disease, and underlying malignancy are more prevalent among nonsurvivors of necrotizing fasciitis compared to survivors.^17^, ^21^, ^24^, ^25^ Existing literature studying necrotizing fasciitis of the H&N is comprised mostly of small case series with limited consensus on risk factors or clinical markers predictive of poor outcomes. Further, current prognostic scores for necrotizing fasciitis are limited in terms of the availability of clinical data for score calculation, as well as in reliability and correlation with clinical outcomes in H&N necrotizing fasciitis.^12^, ^16^, ^17^, ^18^, ^19^, ^20^, ^21^, ^22^, ^26^, ^27^

This case series of 33 patients with H&N necrotizing fasciitis analyzes clinical data to establish patient characteristics associated with poor outcomes, specifically death and DNM, in H&N necrotizing fasciitis. Furthermore, the present study proposes an abbreviated SOFA score for necrotizing fasciitis (nfSOFA) associated with poor outcomes.

## Methods

Following approval from the Vanderbilt University Institutional Review Board (IRB #181591), a single‐institution database was queried using International Classification of Diseases (ICD)‐9 (728.86) and ICD‐10 (M72.6) codes to identify cases of necrotizing fasciitis (N = 1213). A focused chart review resulted in 399 confirmed cases of necrotizing fasciitis between 2006 and 2021; of these, 33 involved the H&N. To be included in the study, patients had to be at least 18 years of age and have both a pathology report and operative findings consistent with necrotizing fasciitis.

A retrospective chart review was then used to collect patient demographics, comorbidities, laboratory data, surgical interventions, and outcomes. Using admission labs, respiratory status, and past medical history, an abbreviated nfSOFA was calculated for each patient, incorporating platelets and creatinine as well as the need for mechanical ventilation and the presence of diabetes (Supplemental Table S1, available online). The same point cutoffs as those used in the complete SOFA score were used to assign points for platelets and creatinine values.^28^ Presence of diabetes and the need for mechanical ventilation were scored as binary variables, with 1 point each assigned for these factors.

**Figure 1:**
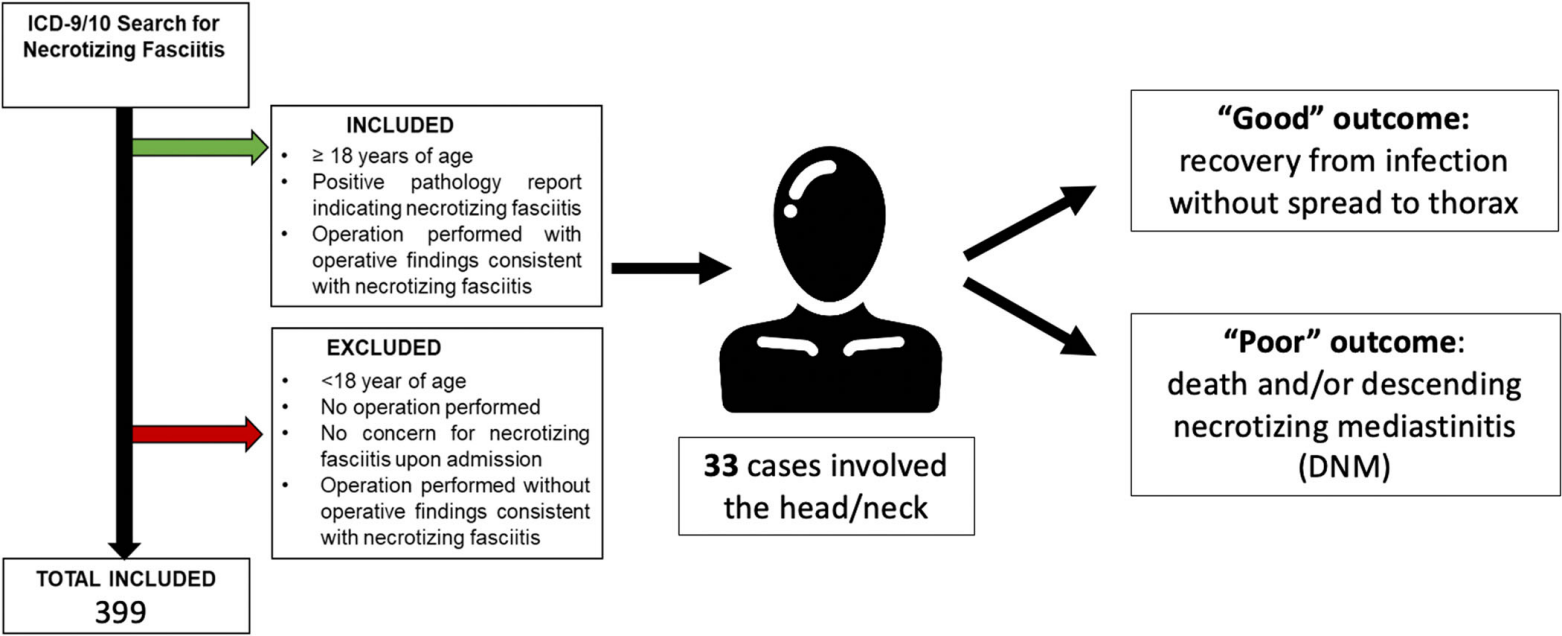
Schematic of inclusion and/or exclusion criteria and “poor” versus “good” outcomes group definitions. ICD, International Classification of Diseases.

Within the H&N cohort, patients were sorted into groups based on outcomes, with the “poor” outcomes group defined by death or spread of infection to the thorax (DNM). Patients who survived their infections without spreading to the thorax comprised the “good” outcomes group (Figure 1). Comparisons between groups were made with Mann‐Whitney *U* tests for continuous variables, as well as Fisher's exact and *χ* ^2^ tests for categorical variables where appropriate. Multivariate analysis was not possible due to the small sample size in this study population.

## Results

### Demographics

From the total study cohort of 399 patients with confirmed necrotizing fasciitis, 33 patients had necrotizing fasciitis involving the H&N. The H&N cohort included 18 men (54.5%) and 15 women (45.5%) with a median age of 52 years (range 19‐75 years). Twenty‐five patients (75.8%) presented as transfers from outside hospitals. The most common known mechanism of infection was odontogenic (12 patients, 36.4%), followed by infection from a traumatic injury (4 patients, 12.1%). Twelve patients (36.4%) had H&N necrotizing fasciitis infections of unknown cause. At the time of presentation, 42.4% of patients had at least 2 medical comorbidities, the most common of which was obesity (Supplemental Table S2, available online).

### H&N Versus Other Anatomic Regions

Comparing the H&N cohort to necrotizing fasciitis of all other anatomic regions, there was no difference in biological sex, age, or length of stay between groups. There were, however, significant differences in overall mortality as well as the prevalence of obesity and hypertension between groups. In the H&N group, 2 patients (6.08%) died as a result of their infections, compared to a 20.8% mortality rate in the non‐H&N group (*p* = .041). There were also lower rates of both obesity and hypertension in the H&N group compared to the non‐H&N group. In the H&N group, 27.3% of patients had a BMI that qualified them as obese, compared to 63.7% in the non‐H&N group (*p* < .001). Additionally, 42.4% of patients in the H&N group had a known diagnosis of hypertension, compared to 60.9% in the non‐H&N group (*p* = .038). There was no difference in the prevalence of diabetes, cancer, heart disease, liver disease, kidney disease, or peripheral vascular disease between groups (*p* > .05).

### Microbiology

**Figure 2:**
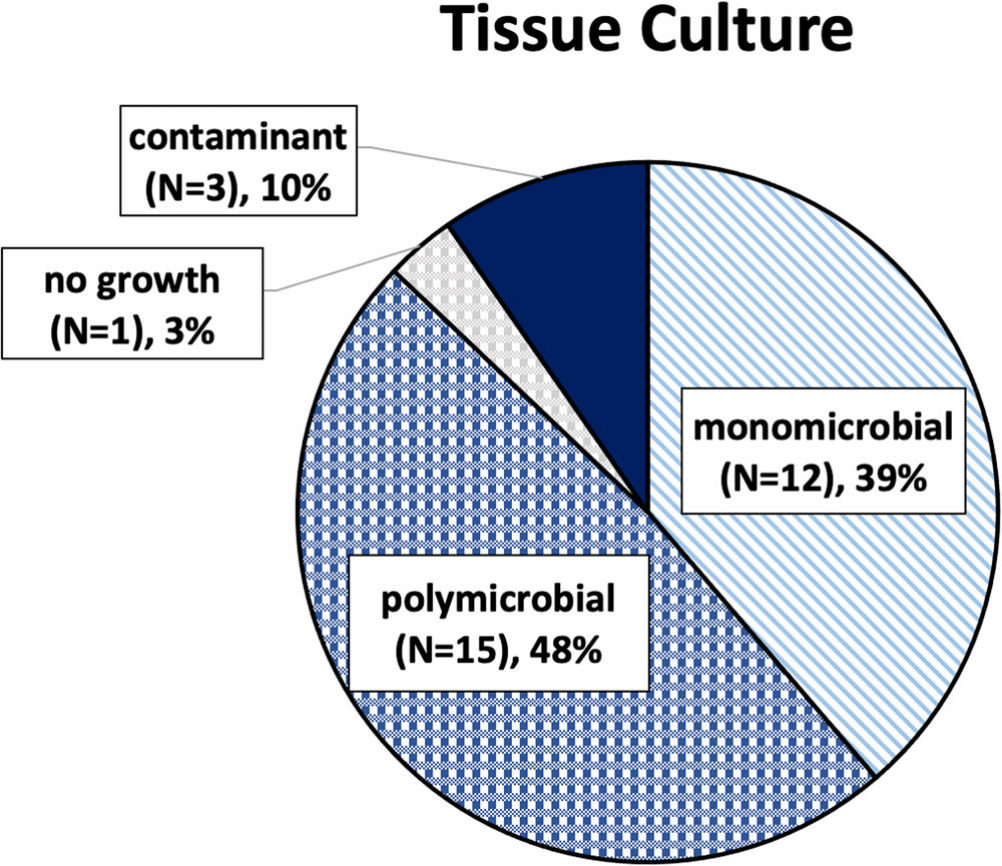
All available cultures from infected tissues by infection type (mono‐ vs polymicrobial).

**Figure 3:**
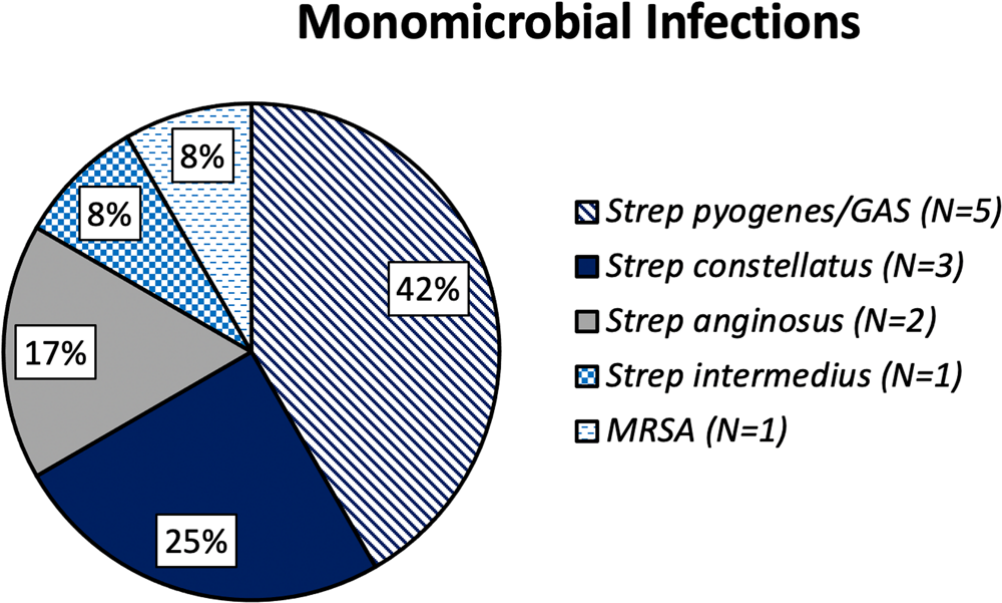
All monomicrobial infections by individual pathogen. GAS, group A streptococcus; MRSA, methicillin‐resistant *Staphylococcus aureus*.

**Figure 4:**
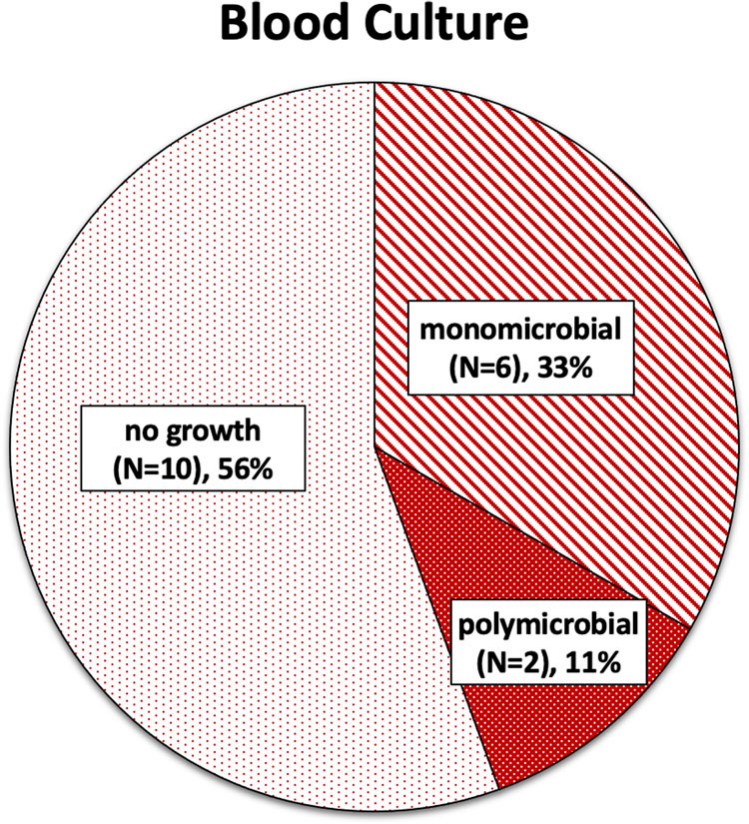
All available blood cultures by infection type (mono‐ vs polymicrobial).

Within the H&N cohort, tissue culture data were available for 31 of 33 patients (93.9%). One patient died prior to the finalization of their tissue culture results, and another patient's results were unavailable due to a transfer from an outside institution. Of all available tissue cultures, 48% were polymicrobial, 40% were monomicrobial, and 1 showed no growth on tissue culture (Figure 2). Tissue cultures from monomicrobial infections predominantly resulted with *Streptococcus pyogenes*, or group A strep (Figure 3). Of the 12 monomicrobial infections, 11 infections (91.7%) were due to *Streptococcus* species, and 1 (8.3%) was due to methicillin‐resistant *Staphylococcus aureus* (Figure 3). Blood culture data were available for 18 of the 33 patients (54.5%). On blood culture, 6 patients (33.3%) had monomicrobial findings, 2 patients (11.1%) had polymicrobial findings, and 10 patients (55.6%) exhibited no growth (Figure 4, Supplemental Table S3, available online).

### Outcomes

Eight patients (24.2%) with confirmed H&N necrotizing fasciitis required tracheostomy, and 9 additional patients (27.3%) required intubation, for a total of 17 patients (51.5%) who required respiratory intervention and mechanical ventilation. The median number of total debridements per patient was 4 (range 1‐12). Eight patients (24.2%) experienced a spread of infection to the mediastinum, and 2 patients (6.1%) did not survive their infections. Details regarding surgical management for each patient can be found in Supplemental Table S4, available online.

Patients whose infections resulted in either DNM or death comprised the “poor” outcomes group, and patients who survived their infections without spreading to the thorax comprised the “good” outcomes group. There was no significant difference in the number of medical comorbidities in the “poor” outcomes group (median: 3; range 0‐5) compared to the “good” outcomes group (median: 1; range 0‐5) (*p* = .179). In the “poor” outcomes group, 7 patients (77.8%) had a diagnosis of type 2 diabetes mellitus, compared to 8 of 24 (33.3%) patients in the “good” outcomes group (*p* = .047). Median A1C in the “poor” outcomes group was 9.2 (range 5.3‐13.8) compared to the median A1C of 5.7 (range 4.4‐14.1) in the “good” outcomes group (*p* = .058). When only those patients with a clinical diagnosis of diabetes were compared between groups, the median A1C in the “good” outcomes group was 6 (n = 7; range 4.4‐14.1) (*p* = .187).

Regarding respiratory intervention, 8 of 9 patients (88.9%) in the “poor” outcomes group required intubation or tracheostomy, compared to 9 of 24 patients (37.5%) in the “good” outcomes group (*p* = .017). The difference in total debridements performed between groups did not reach our predetermined cutoff for statistical significance. The was a median of 5 debridements in the “poor” outcomes group (range 1‐12) and a median of 3 debridements in the “good” outcomes group (range 1‐7) (*p* = .067) (Supplemental Table S5, available online).

### LRINEC Score

The LRINEC score, designed to distinguish necrotizing fasciitis from other soft tissue cellulitis, requires C‐reactive protein(CRP), white blood cells, hemoglobin, sodium, glucose, and creatinine for calculation.^13^ CRP values were available upon admission for 19 of 33 patients (57.6%). CRP values at any point throughout hospitalization were available for 24 of 33 patients (71.7%). Among the 24 patients with H&N necrotizing fasciitis and laboratory data sufficient to calculate the LRINEC score at any point throughout hospitalization, the median LRINEC score was 6 (mean: 5.42; range 0‐10). There was no difference in LRINEC scores between the “poor” outcomes group (n = 7; median: 8; mean: 6.57; range 1‐8) and the “good” outcomes group (n = 15; median: 6; mean: 5.80; range 0‐10) (*p* = .418).

### Abbreviated nfSOFA

Using admission labs, respiratory status, and diabetes history, an abbreviated nfSOFA was calculated for each patient (Supplemental Tables S1 and S2, available online). The median nfSOFA for the “poor” outcomes group (defined by death or DNM) was 2 (range 1‐5), while the median nfSOFA for the “good” outcomes group (survival without infection spread) was 1 (range 0‐6). Eight of 9 patients (88.9%) in the “poor” outcomes group had a nfSOFA of 2 or greater, while only 9 patients (37.5%) in the “good” outcomes group had a nfSOFA of 2 or greater (*p* = .017). In other words, in our cohort, patients with a nfSOFA score of 2 or greater were significantly more likely to be in the “poor” outcomes group.

## Discussion

The present study reports a retrospective case series of 33 patients with surgically confirmed necrotizing fasciitis of the H&N—one of the largest single institution cohorts reported in the literature to date. In this study population, necrotizing fasciitis of the H&N was distinct from necrotizing fasciitis of other anatomic regions, with a lower mortality rate as well as a lower prevalence of obesity and hypertension. Additionally, within the H&N cohort, an abbreviated nfSOFA of 2 or greater was associated with worse outcomes, specifically death and DNM.

There are few other case series reporting 30 or more patients with necrotizing fasciitis of the H&N.^24^, ^26^, ^29^, ^30^, ^31^ Zhang et al report a case series of 27 patients with DNM,^9^ and numerous other authors have reported smaller case series of H&N necrotizing fasciitis.^3^, ^4^, ^5^, ^16^, ^19^, ^23^, ^25^, ^27^, ^32^, ^33^ An additional strength of this data set is its stringent inclusion criteria, which required both tissue pathology and operative findings consistent with necrotizing fasciitis for inclusion.

The mortality rate from H&N necrotizing fasciitis in this study was 6.1%, consistent with prior case series by Mathieu et al^31^ and Lanisnik and Čizmarevič,^29^ and lower than many previously reported mortality rates for H&N necrotizing fasciitis.^4^, ^17^, ^24^, ^32^, ^34^ This lower mortality rate may be attributable to more aggressive surgical management, as the median number of debridements was 4, higher than previously reported averages,^4^, ^24^, ^34^ but similar to the number of debridements reported by Ferzli et al (mean 4.6), which likewise corresponded to a lower mortality rate at 10%.^32^ Wolf et al reported a similarly low mortality rate of H&N necrotizing fasciitis; however, patients in that study had relatively few medical comorbidities.^23^ In the present study, patients had a much higher prevalence of comorbidities, with 42.4% of patients having at least 2 medical comorbidities at the time of admission. Notably, the study by Wolf and colleagues took place in Denmark, compared to the present study based in the southeastern United States, where obesity and type II diabetes have been shown to cluster with highest prevalence.^35^ Fifteen patients (45.5%) in the present study had diabetes, and significantly more patients in the “poor” outcomes group had diabetes compared to the “good” outcomes group. This is consistent with prior studies reporting a greater prevalence of diabetes among nonsurvivors of necrotizing fasciitis compared to survivors.^17^, ^31^, ^36^ Notably, however, a recent large systematic review reported no relationship between diabetes and DNM.^34^ Prior studies have reported a higher prevalence of active malignancy among nonsurvivors of necrotizing fasciitis compared to survivors.^24^, ^30^ However, only 2 patients in the present study were known to have underlying malignancy. One of these patients experienced a spread of infection to the thorax and ultimately died as a result of sepsis and declining respiratory function, and the other survived their infection without spreading to the thorax.

Regarding microbiology, the proportions of monomicrobial and polymicrobial infections in this study are generally consistent with prior data, though the relative frequencies vary widely across studies.^17^, ^21^, ^37^, ^38^, ^39^, ^40^, ^41^ Tissue cultures from monomicrobial, or type II, infections predominantly resulted with *Streptococcus pyogenes*, or group A strep (Figure 3), consistent with prior literature.^2^, ^36^, ^37^, ^40^ Only 1 patient's tissue cultures showed no growth, and this patient had been started on antibiotics at an outside hospital prior to obtaining tissue cultures at our institution.

This is the first study to our knowledge that compares H&N necrotizing fasciitis with necrotizing fasciitis of other anatomic regions cared for at the same institution. The H&N cohort had a lower mortality rate as well as a lower prevalence of obesity and hypertension. Given that there was no difference in the prevalence of diabetes, cancer, heart disease, liver disease, kidney disease, or peripheral vascular disease between groups, it is unlikely that the difference in the prevalence of obesity and hypertension between groups accounts for the difference in mortality rate. Rather, we hypothesize that patients with necrotizing infections involving the H&N are more likely to take notice of their infections and seek care earlier compared to patients with infections involving the trunk or extremities. However, it is challenging to test this theory, as 25 patients (75.8%) in the H&N cohort presented as transfers from outside hospitals without definitive data on time to presentation available in retrospective chart review. A future prospective study and focused data collection on time to presentation would be needed to address this hypothesis.

Prior studies investigating prognostic markers for necrotizing fasciitis of the H&N have focused on distinguishing necrotizing fasciitis from other soft tissue infections using scores such as the LRINEC score.^12^, ^16^, ^18^, ^19^, ^20^ Reliability of the LRINEC score remains variable across necrotizing fasciitis of all anatomic regions.^12^, ^16^, ^17^, ^18^, ^19^, ^20^, ^21^, ^22^ Others have studied well‐established critical care scores, such as the Acute Physiology and Chronic Health Evaluation (APACHE) II^26^ and quick sequential organ failure assessment (qSOFA)^27^ as they relate to morbidity and mortality in H&N necrotizing fasciitis. In a study by Elander et al, there was no difference in APACHE II scores between survivors and nonsurvivors of H&N NF.^26^ In a study by LaMothe et al of patients with H&N NF, only 3 of 10 patients had a qSOFA score greater than 2, and this was not reported to correlate with patient outcomes.^27^

The abbreviated nfSOFA presented in this study tailors the SOFA score to include specific but readily available data relevant to necrotizing fasciitis outcomes (Supplemental Table S1, available online). Unlike the complete SOFA score—which requires CBC, CMP, arterial blood gas, and data on hemodynamics—or the LRINEC score—which requires CBC, BMP, and CRP—the nfSOFA only requires data available from a CBC and BMP. Such simplicity, which lends the nfSOFA score to easier clinical implementation, also sacrifices granular details that may better contextualize prognosis. Given that the nfSOFA score has relatively few components, aberrancy in any 1 component may inappropriately weight the score and incorrectly estimate a patient's risk for poor outcome. For example, in the current scoring system, an elevated creatinine of 2.0 or greater alone would lead to a nfSOFA score of 2, which was shown to correlate with poor outcomes in this study. However, only 1 patient in the poor outcomes group had an elevated creatinine in the absence of other points in the nfSOFA system, and, furthermore, creatinine elevations reflective of acute or chronic kidney disease have important implications for wound healing and infection recovery.^17^, ^42^, ^43^, ^44^

Further adjustment of the nfSOFA score utilizing a larger, multi‐institutional cohort will be necessary. In the current model, the presence of diabetes and the need for mechanical ventilation are scored as binary variables, with 1 point each assigned for these factors. This system likely undervalues the clinical impact of diabetes and intubation and/or tracheostomy requirement on H&N necrotizing fasciitis, as the need for intubation alone has been shown to correlate with mortality in sepsis of all causes.^45^ The rationale for including these elements as binary values was to avoid artificially inflating scores without sufficient data to properly weight each factor; however, future studies should consider adjusting relative weights of the nfSOFA score components to best reflect the clinical impact of these variables.

Regarding limitations, this study is limited foremost by its retrospective nature and therefore its reliance on ICD‐9 and ICD‐10 codes for identification of cases. For this reason, only cases identified as necrotizing fasciitis via electronic coding were identified, and only those cases with operative and pathology reports consistent with necrotizing fasciitis were included. This methodology makes it likely that many cases of H&N necrotizing fasciitis were missed in our search strategy due to imprecise or incomplete coding. Additionally, this study included patients who received care over a 15‐year period between 2006 and 2021. Trends in diagnosis and treatment for necrotizing fasciitis have evolved over time, and these changes in practice patterns were unable to be controlled given the small sample size. Further, because this study of a rare disease process was conducted at a single institution, the sample size was also insufficient to conduct multivariate analyses. Future studies may consider analyzing across institutions to obtain a larger sample size of patients to better address confounding variables via multivariate analyses. Finally, due to the limited availability of data to calculate complete SOFA and LRINEC scores for each patient, comparative analytics between these metrics and the nfSOFA score is not possible in the present study. This may be an area for future investigation, especially in a prospective setting in which all necessary clinical data can be obtained to calculate each score for a sufficient number of patients. Future prospective studies will also be required for formal validation of the nfSOFA score proposed herein.

Ultimately, necrotizing fasciitis remains an emergent condition that relies upon experienced clinical acumen for diagnosis and urgent surgical debridement for management. A prognostic score such as the nfSOFA may be helpful for communication—both between providers as well as between providers and patient families—during the early stages of care for patients with H&N necrotizing fasciitis.

## Conclusion

Necrotizing fasciitis of the H&N is clinically distinct from necrotizing fasciitis of other anatomic regions, with a lower mortality rate and lower prevalence of obesity and hypertension. Patients with H&N necrotizing fasciitis whose infections led to DNM or death were more likely to have diabetes compared to patients who survived without infection spread. An abbreviated nfSOFA—which includes platelet count, creatinine, diabetes, and respiratory status—of 2 or greater was associated with poor outcomes, specifically death and DNM.

## Author Contributions

**Kelly L. Vittetoe**, study design, data collection, data analysis, manuscript composition; **Samuel R. Johnson**, data collection, data analysis, manuscript review; **Teresa A. Benvenuti**, data collection, manuscript review; **Jonathan G. Schoenecker**, study design/oversight, manuscript review; **Stephanie N. Moore‐Lotridge**, study design/oversight, data analysis, manuscript review; **Sarah L. Rohde**, study design/oversight, manuscript review.

## Disclosures

### Competing interests

None.

### Funding source

None.

## Supporting information

Supplemental Material.
