# Supplementary material for: Head and Neck Necrotizing Fasciitis: Abbreviated SOFA Score Associated With Death and Infection Spread
Source: OTO Open. 2023 Aug 9;7(3):e68. doi: 10.1002/oto2.68 (PMC10410339; doi:10.1002/oto2.68)
Supplement: Supplementary file 1 — Supplemental Material. [file OTO2-7-e68-s001.docx]

| **Category** |  | **Point Value** |
| --- | --- | --- |
| **Mechanical Ventilation** | No | 0 |
|  | Yes | 1 |
| **Diabetes** | No | 0 |
|  | Yes | 1 |
| **Platelets (x10^3^/µL)** | ≥ 150 | 0 |
|  | 100 – 149 | 1 |
|  | 50 – 99 | 2 |
|  | 20 – 49 | 3 |
|  | <20 | 4 |
| **Creatinine (mg/dL)** | <1.2 | 0 |
|  | 1.2 – 1.9 | 1 |
|  | 2.0 – 3.4 | 2 |
|  | 3.5 – 4.9 | 3 |
|  | ≥5.0 | 4 |

**Supplemental Table 1.** Abbreviated SOFA Score for Necrotizing Fasciitis (nfSOFA)

**Supplemental Table 2.** Individual clinical details for each patient in the head and neck cohort

| **Record** | **Sex** | **Age** | **PMH** | **Mechanism of Infection** | **LOS (days)** | **WBC** | **Cr** | **Plt** | **CRP** | **LRINEC** | **nfSOFA Score** | **Tissue Culture** |
| --- | --- | --- | --- | --- | --- | --- | --- | --- | --- | --- | --- | --- |
| 1 | M | 49 | obesity, DM, CAD, HTN, cancer | odontogenic | 5 | 15 | 3.66 | 268 | 437.2 | 9 | 5 | *staph epidermidis* |
| 2 | M | 65 | DM, CAD, HTN | odontogenic | 1 | 24.7 | 1.23 | 158 |  |  | 3 | No final results at time of death |
| 3 | F | 59 | DM, CAD, HTN | Peritonsillar abscess | 24 | 10 | 0.55 | 183 | 233.1 | 7 | 2 | *strep viridans, candida glabrata, strep constellatus, strep anginosus* |
| 4 | F | 25 | obesity, DM | idiopathic (neck abscess) | 12 | 10 | 0.58 | 181 | 174.3 | 8 | 2 | *strep constellatus* |
| 5 | M | 22 | none | odontogenic | 29 | 21.7 | 0.79 | 352 | 103 | 1 | 2 | *strep anginosus* |
| 6 | F | 55 | DM, CAD, HTN | trauma | 27 | 14.9 | 1.77 | 193 | 442.4 | 9 | 1 | *strep pyogenes, MRSA* |
| 7 | F | 44 | obesity, DM, HTN | odontogenic | 31 | 15.3 | 1.83 | 315 |  |  | 3 | *strep constellatus* |
| 8 | F | 25 | none | idiopathic (neck abscess) | 24 | 31.1 | 0.74 | 219 | 206.8 | 7 | 2 | *staph epidermidis* |
| 9 | M | 58 | DM | infected sebaceous cyst | 11 | 26 | 1.35 | 205 | 116.9 | 9 | 3 | No available culture data |
| 10 | M | 75 | obesity, DM, CKD, CAD, HTN | odontogenic | 33 | 3.4 | 2.91 | 272 | 297.6 | 9 | 4 | *strep intermedius, candida albicans* |
| 11 | M | 60 | DM, CAD, HTN, PVD | odontogenic | 12 | 21.8 | 2.32 | 86 | 157 | 9 | 6 | *proteus, strep pyogenes, enterococcus faecalis* |
| 12 | M | 48 | DM | odontogenic | 5 | 20 | 121 | 502 |  |  | 1 | *strep constellatus* |
| 13 | F | 51 | obesity, HTN | idiopathic (neck abscess) | 6 | 16.1 | 1.77 | 250 | 296 | 8 | 1 | *strep anginosus* |
| 14 | M | 19 | obesity, HTN, DM | optic origin | 5 | 6 | 1.14 | 163 | 72.1 | 0 | 0 | no growth; started on antibiotics at outside hospital prior to transfer |
| 15 | M | 59 | HCC/cirrhosis, CAD, HTN | trauma | 11 | 27.6 | 0.68 | 138 | 217.6 | 8 | 1 | *MRSA, staph epidermidis, strep pyogenes, Enterobacter cloacae* |
| 16 | F | 59 | none | optic origin | 13 | 12.9 | 1.18 | 104 |  |  | 1 | *MRSA, strep pyogenes* |
| 17 | F | 62 | obesity, HTN | odontogenic | 9 | 12.5 | 1.16 | 437 | 233.3 | 6 | 0 | *strep anginosus, prevotella denticola, candida albicans, candida tropicalis* |
| 18 | M | 40 | none | odontogenic | 9 | 14.3 | 0.73 | 251 |  |  | 0 | *strep anginosus, strep constellatus* |
| 19 | F | 24 | none | post-op thyroidectomy | 24 | 15 | 1.14 | 311 | 88.7 | 3 | 2 | *Strep pyogenes, MRSA* |
| 20 | F | 43 | none | odontogenic | 19 | 12.1 | 0.44 | 81 | 70.1 | 1 | 4 | *MRSA, candida albicans, staph epidermidis* |
| 21 | F | 52 | obesity, DM, CAD, HTN | idiopathic (neck abscess) | 24 | 24.7 | 0.95 | 209 |  |  | 2 | *strep constellatus* |
| 22 | F | 47 | CAD | odontogenic | 14 | 38 | 2.19 | 164 | 261.1 | 9 | 4 | *strep viridans, candida albicans, candida tropicalis* |
| 23 | F | 73 | none | idiopathic (periorbital cellulitis) | 22 | 10.2 | 1.01 | 199 | 238.5 | 6 | 2 | *strep pyogenes* |
| 24 | F | 45 | none | odontogenic | 19 | 39.4 | 0.47 | 307 | 286.4 | 10 | 2 | *strep intermedius (alpha)* |
| 25 | M | 56 | none | idiopathic (periorbital cellulitis) | 10 | 26.2 | 1.47 | 256 | 312.4 | 7 | 3 | *strep pyogenes* |
| 26 | M | 54 | DM | trauma | 7 | 5.9 | 0.45 | 236 |  |  | 0 | *staph epidermidis* |
| 27 | M | 47 | DM | idiopathic (neck abscess) | 22 | 20.4 | 1.18 | 389 | 296 | 8 | 0 | *MSSA, strep agalactiae* |
| 28 | M | 76 | HTN | idiopathic (periorbital cellulitis) | 7 | 34.6 | 0.83 | 130 | 341.1 | 8 | 1 | *strep pyogenes* |
| 29 | M | 72 | HTN | idiopathic (periorbital cellulitis) | 15 | 13 | 0.68 | 340 | 196.9 | 7 | 0 | *Strep intermedius, staph epidermidis* |
| 30 | M | 48 | obesity, HTN, CKD, DM | idiopathic (neck abscess) | 9 | 26.7 | 0.88 | 405 | 360.7 | 9 | 0 | *MRSA* |
| 31 | F | 57 | none | idiopathic (neck abscess) | 7 | 17 | 0.53 | 277 |  |  | 0 | *strep constellatus, staph epidermidis (MRSE)* |
| 32 | M | 27 | none | idiopathic (facial abscess) | 5 | 38.1 | 1.24 | 347 | 147.3 | 5 | 1 | *strep constellatus, strep intermedius (alpha), fusobacterium necrophorum* |
| 33 | M | 56 | HTN | trauma | 30 | 17.9 | 1.58 | 248 |  |  | 1 | *strep pyogenes* |

CAD: coronary artery disease; CKD: chronic kidney disease; DM: diabetes mellitus; HTN: hypertension; PVD: peripheral vascular disease. LOS: length of stay; WBC: white blood cell count; Cr: creatinine; Plt: platelet count; CRP: C-reactive protein.

**Supplemental Table 3.** From all tissue culture data, incidence of infection by pathogen, organized by genus and species. Note that these data are from both monomicrobial and polymicrobial infections.

| Genus | Species | N |
| --- | --- | --- |
| **Streptococcus** |  |  |
|  | *pyogenes (Group A)* | 9 |
|  | *constellatus* | 8 |
|  | *anginosus* | 5 |
|  | *intermedius* | 4 |
|  | *agalactiae (Group B)* | 1 |
|  |  |  |
| **Staphylococcus** |  |  |
|  | *epidermidis/coagulase negative* | 7 |
|  | *aureus (MRSA)* | 5 |
|  | *aureus (MSSA)* | 1 |
|  |  |  |
| **Candida** |  |  |
|  | *albicans* | 4 |
|  | *tropicalis* | 2 |
|  | *glabrata* | 1 |
|  |  |  |
| **Other** |  |  |
|  | *Prevotella denticola* | 1 |
|  | *Proteus miribalis* | 1 |
|  | *Enterococcus faecalis* | 1 |
|  | *Enterobacter cloacae* | 1 |
|  | *Fusobacterium necrophorum* | 1 |

**Supplemental Table 4.** Individual management details for each patient in head and neck cohort

| **Record** | **Sex** | **Age** | **Infection Spread** | **Outcome** | **Airway Management** | **Number of Debridements** | **Wound Vaccuum Placement** | **Surgical Management** |
| --- | --- | --- | --- | --- | --- | --- | --- | --- |
| 1 | M | 49 | DNM | Death | Tracheostomy | 3 | Yes | Last case aborted due to worsening respiratory status and inability to tolerate operation |
| 2 | M | 65 | None | Death | Intubation | 1 | No | Radical resection of right facial and neck soft tissues, parotid gland, facial nerve, masseter and medial pterygoid muscles, submandibular gland, facial musculature |
| 3 | F | 59 | DNM | Survived | Tracheostomy | 8 | Yes | Right pectoralis major muscle flap, STSG |
| 4 | F | 25 | DNM | Survived | Intubation | 5 | Yes | Delayed primary closure |
| 5 | M | 22 | DNM | Survived | Tracheostomy | 6 | Yes | Delayed primary closure |
| 6 | F | 55 | DNM | Survived | No Intervention | 3 | No | Initial primary closure, later bilobed pedicle flap on right lateral palpebral artery with pedicle flap on left lateral palpebral artery |
| 7 | F | 44 | DNM | Survived | Tracheostomy | 12 | Yes | Left latissimus dorsi muscle free flap to left transverse cervical artery / vein |
| 8 | F | 25 | DNM | Survived | Tracheostomy | 4 | No | Delayed primary closure |
| 9 | M | 58 | DNM | Survived | Intubation | 6 | Yes | STSG thigh donor to right chest |
| 10 | M | 75 | None | Survived | Intubation | 2 | Yes | Closure of pharyngotomy with mylohyoid rotational flap |
| 11 | M | 60 | None | Survived | Intubation | 7 | No | Buccal advancement flap, delayed primary closure |
| 12 | M | 48 | None | Survived | No Intervention | 2 | No | Delayed primary closure |
| 13 | F | 51 | None | Survived | No Intervention | 1 | No | Primary closure |
| 14 | M | 19 | None | Survived | No Intervention | 1 | No | Primary closure |
| 15 | M | 59 | None | Survived | No Intervention | 3 | No | PMFF |
| 16 | F | 59 | None | Survived | No Intervention | 4 | No | Delayed primary closure |
| 17 | F | 62 | None | Survived | No Intervention | 5 | No | Delayed primary closure |
| 18 | M | 40 | None | Survived | No Intervention | 2 | No | Delayed primary closure |
| 19 | F | 24 | None | Survived | Tracheostomy | 7 | Yes | STSG |
| 20 | F | 43 | None | Survived | Tracheostomy | 5 | No | Delayed primary closure |
| 21 | F | 52 | None | Survived | Tracheostomy | 2 | No | Delayed primary closure |
| 22 | F | 47 | None | Survived | Intubation | 4 | No | Delayed left neck rotation advancement flap |
| 23 | F | 73 | None | Survived | Intubation | 6 | No | STSG |
| 24 | F | 45 | None | Survived | Intubation | 7 | Yes | Local fasciocutaneous advancement flap of anterior neck, abdominal/dermal fat graft for submental wound closure |
| 25 | M | 56 | None | Survived | Intubation | 3 | No | Delayed primary closure, delayed STSG |
| 26 | M | 54 | None | Survived | No Intervention | 1 | No | Primary closure, delayed need for scalp tissue expanders and advancement flap |
| 27 | M | 47 | None | Survived | No Intervention | 6 | Yes | Vertical trapezius musculocutaneous flap reconstruction |
| 28 | M | 76 | None | Survived | No Intervention | 2 | No | Delayed primary closure |
| 29 | M | 72 | None | Survived | No Intervention | 3 | No | Delayed primary closure |
| 30 | M | 48 | None | Survived | No Intervention | 4 | Yes | Delayed primary closure |
| 31 | F | 57 | None | Survived | No Intervention | 3 | Yes | FTSG (contralateral supramammary) |
| 32 | M | 27 | None | Survived | No Intervention | 2 | No | Delayed primary closure |
| 33 | M | 56 | None | Survived | No Intervention | 4 | No | Delayed primary closure |

DNM: descending necrotizing mediastinitis; STSG: split thickness skin graft; FTSG: full thickness skin graft; PMFF: paramedian forehead flap.

**Supplemental Table 5.** Comparison of Clinical Factors Between “Good” and “Poor” Outcomes Groups

|  | **“Good” Outcomes**  **(N = 24; 72.7%)** | **“Poor” Outcomes (Death, DNM)**  **(N = 9; 27.3%)** | **p-value** |
| --- | --- | --- | --- |
| Reported Comorbidities | Median = 3  (Range: 0-5) | Median = 5  (Range 0-5) | 0.179 |
| Diabetes Diagnosis | N = 8 (33.3%) | N = 7 (77.8%) | **0.047** |
| Hemoglobin A1C on Admission | Median = 5.7  (Range: 4.4-14.1) | Median = 9.2  (Range 5.3–13.8) | 0.058 |
| Respiratory Intervention (Intubation, Tracheostomy) | N = 9 (37.5%) | N = 8 (88.9%) | **0.017** |
| Surgical Debridements | Median = 3  (Range: 1-7) | Median = 5  (Range: 1-12) | 0.067 |
| LRINEC Score | Median = 6  (Range: 0-10) | Median = 8  (Range: 1-8) | 0.418 |
| nfSOFA Score ≥ 2 | N = 9 (37.5%) | N = 8 (88.9%) | **0.017** |
